# Supplementary material for: Molecular basis of ALK1-mediated signalling by BMP9/BMP10 and their prodomain-bound forms
Source: Nat Commun. 2020 Apr 1;11:1621. doi: 10.1038/s41467-020-15425-3 (PMC7113306; doi:10.1038/s41467-020-15425-3)
Supplement: Supplementary file 1 — Supplementary Information [file 41467_2020_15425_MOESM1_ESM.pdf]

# Molecular basis of ALK1-mediated signalling by BMP9/BMP10 and their prodomain-bound forms

Salmon et al.

<u>**Supplementary Table and Figures**</u>

Supplementary Table 1. Data collection and refinement statistics

Supplementary Figure 1. Schematic and nomenclature of BMP9 and BMP10

Supplementary Figure 2. Comparison of the 2.3 Å and 2.8 Å BMP10:ALK1 crystal structures

Supplementary Figure 3. Stereo image of an electron density map of the 2.3 Å BMP10:ALK1 structure

Supplementary Figure 4. Sequence alignment of human ALK1, ALK2, ALK3 and ALK6 extracellular domains

Supplementary Figure 5. Sequence alignment of full-length human proBMP9 and proBMP10

Supplementary Figure 6. Prodomain and GF-domain interface in the pro-BMP9:ALK1 structure

Supplementary Figure 7. Sequence coverage in the pro-BMP9:ALK1 structure

Supplementary Figure 8. ALK1 ECD can form complexes with pro-BMP9 and pro-BMP10 without displacing the prodomains

Supplementary Figure 9. *Id1* gene induction in C2C12 cells treated with pro-BMP9 or its mutants

Supplementary Figure 10. Pro-BMP9 D366E mutation selectively lost binding to ALK2

Supplementary Figure 11. Uncropped original Gels and Blots

Supplementary References

## Supplementary Table 1. Data collection and refinement statistics

| | BMP10:ALK1* Crystal 1 PDB code: 6SF3 | BMP10:ALK1* Crystal 2 PDB code: 6SF1 | Pro-BMP9:ALK1* PDB code: 6SF2 |
|---|---|---|---|
| **Data collection** | | | |
| Space group | $P6_522$ | $P6_522$ | $P6_1$ |
| Cell dimensions | | | |
| $a$, $b$, $c$ (Å) | 58.27, 58.27, 311.4 | 57.57, 57.57, 304.06 | 72.61, 72.61, 438.49 |
| $\alpha$, $\beta$, $\gamma$ (°) | 90.00, 90.00, 120.00 | 90.00, 90.00, 120.00 | 90.00, 90.00, 120.00 |
| Resolution (Å) | 311.44-2.30 (2.38-2.30)** | 25.03-2.80 (2.95-2.80)** | 62.88-3.30 (3.48-3.30)** |
| CC half | 0.994 (0.635) | 0.995 (0.862) | 0.997 (0.779) |
| $R_{merge}$ | 0.280 (6.319) | 0.097 (0.519) | 0.154 (1.134) |
| *Mean* $((I) / \sigma(I))$ | 7.4 (1.6) | 9.5 (2.6) | 13.3 (2.8) |
| Completeness (%) | 99.9 (99.8) | 99.7(100) | 100.0 (99.9) |
| Redundancy | 14.7 (15.1) | 4.3 (4.6) | 9.9(10.2) |
| | | | |
| **Refinement** | | | |
| Resolution (Å) | 51.91-2.30 | 24.93-2.80 | 62.25-3.30 |
| No. reflections | 14,963 | 8,108 | 19,518 |
| $R_{work}$ / $R_{free}$ | 0.2140/0.2433 | 0.2441/0.2789 | 0.2426/0.2743 |
| No. atoms | | | |
| Protein | 1446 | 1419 | 4353 |
| Ligand/ion | 0 | 5 | 2 |
| Water | 24 | 5 | 9 |
| $B$-factors (Å$^2$) | | | |
| Protein | 59.1 | 70.3 | 89.4 |
| Ligand/ion | N/A | 101.37 | 127.9 |
| Water | 54.1 | 53.3 | 79.5 |
| R.m.s. deviations | | | |
| Bond lengths (Å) | 0.007 | 0.002 | 0.002 |
| Bond angles (°) | 0.850 | 0.488 | 0.490 |
| Clash score | 3.51 | 3.21 | 4.24 |

* All datasets were obtained from a single crystal.

** Values in parentheses are for highest-resolution shell.

# Supplementary Figure 1

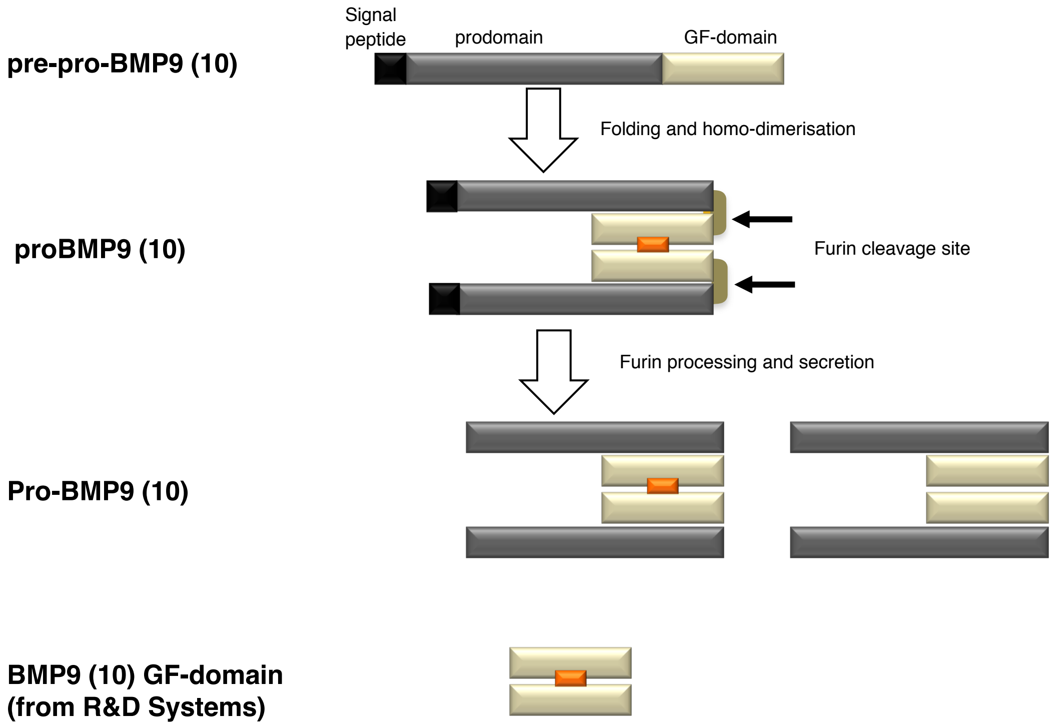


**Supplementary Figure 1.** Schematic and nomenclature of BMP9 and BMP10. The orange block denotes the intermolecular disulfide bond.

# Supplementary Figure 2

**(a)**

**(b)**

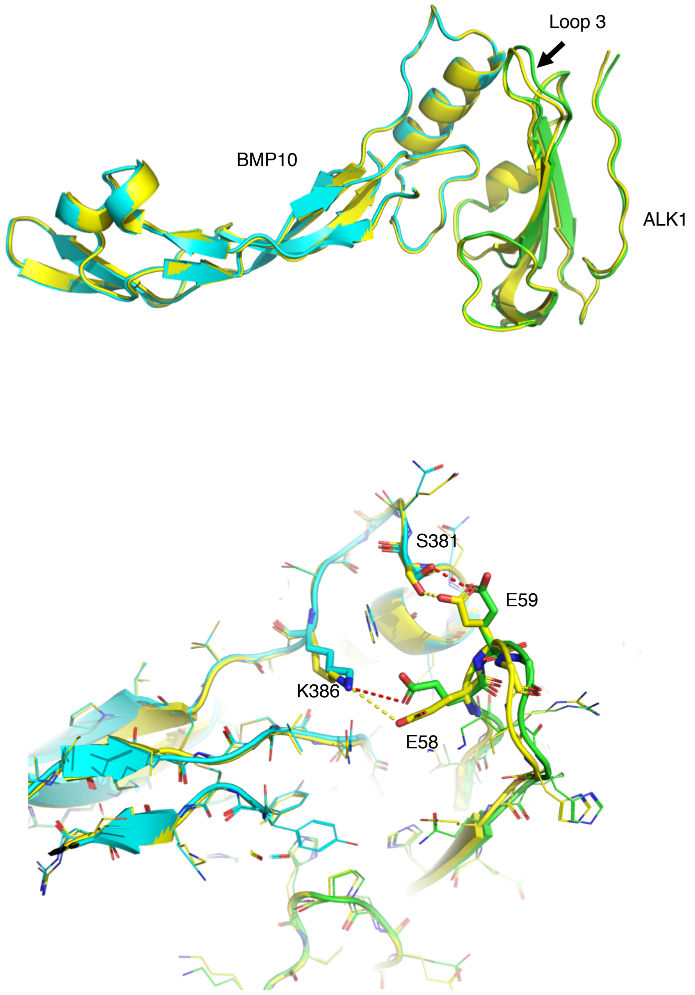


**Supplementary Figure 2.** Comparison of the 2.3 Å (ALK1 in green and BMP10 in cyan) and 2.8 Å (yellow) BMP10:ALK1 crystal structures. Only one monomer of the symmetric dimer is shown. ***(a)*** Two BMP10:ALK1 structures were overlaid. Little difference was detected in the BMP10 region. One minor shift in the ALK1 loop 3 region (arrow) could be observed. Of note, this loop has poor electron density, suggesting flexibility. ***(b)*** A close-up view of the interactions in the ALK1 loop 3 region. Despite of the small changes in the loop position, the interactions between BMP10 and ALK1 are preserved between the two structures. The rest of the side-chains overlay very well throughout the two structures.

# Supplementary Figure 3

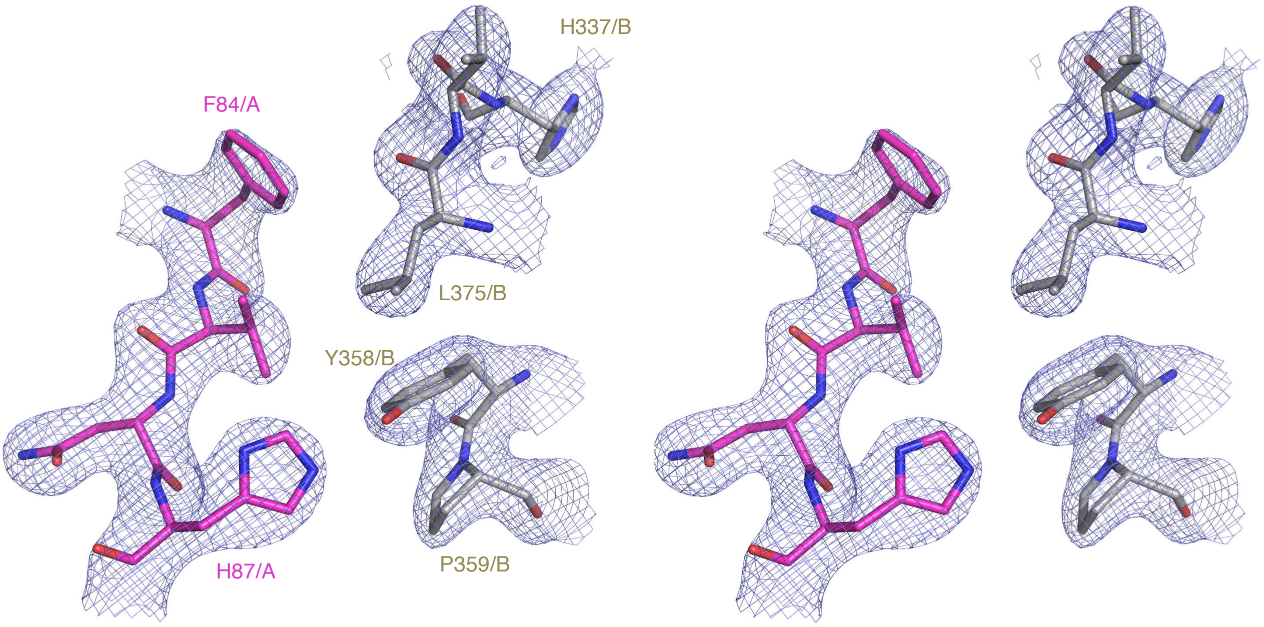


**Supplementary Figure 3.** A stereo image of an electron density map of the 2.3 Å BMP10:ALK1 structure. The 2Fo-Fc electron density (blue, contoured at 1.5 σ) overlaid with the final refined model. ALK1: Magenta, BMP10: grey.

# Supplementary Figure 4

```
                                                    Loop 1
                                               β1   (F1 loop)  β2  Loop 2
ALK1      -------------------------DPVKPSRGPLVTCTCESPHCKG----PTCRGAWCT  52
ALK2      -----------------------MEDEKPKVNPKLYMCVCEGLSCGN---EDHCEGQQCF  54
ALK3      QNLDSMLHGTGMKSDSDQKKSENGVTLAPEDTLPFLKCYCSGHCPDDAINNTCITNGHCF  83
ALK6      -------------------KKEDGESTAPTPRPKVLRCKCHHHCPEDSVNNICSTDGYCF  54

                           Loop 4   α1
               Loop 3     (pre-helix      Loop 5
           β3  (F2 loop) β4  loop)  (3_10 helix)  (F3 loop)  β5
ALK1      VVLVREEGRHPQEHRGCGNLH---RELCRGRPTEF--VNHYCCD-SHLCNHNVSLVLEAT  106
ALK2      SSLSIN-DGFHVYQKGCFQVYEQGKMTCKTPPSPG--QAVECCQ-GDWCNRNITAQLPTK  110
ALK3      AIIEEDDQGETTLASGCMK-YEGSDFQCKDSPKAQLRRTIECCR-TNLCNQYLQPTLPPV  141
ALK6      TMIEEDDSGLPVVTSGCLG-LEGSDFQCRDTPIPHQRRSIECCTERNECNKDLHPTLPPL  113

ALK1      QPPSEQPGTDGQ---  118
ALK2      G--KSFPGTQNFHLE  123
ALK3      VIGPFFDGSIR----  152
ALK6      KNRDFVDGPIHHR--  126
```

**Supplementary** Figure 4. Sequence alignment of human ALK1, ALK2, ALK3 and ALK6 extracellular domains. All ECD sequences were taken from UniProtKB. Secondary structures are labelled over the sequences according to the BMP10:ALK1 structure and the ALK1:BMP9:ActRIIb structure [1].

# Supplementary Figure 5

```
                                                                    Strait jacket
                                                                        α1
ProBMP9     MCPGALWVALP-LLSLLAGSLQGKPLQSWGRGSAGGNAHSPLGVPGGGL-PEHTFNLKMF    58
ProBMP10    --MGSLVLTLCALFCLAAYLVSGSPIMNLEQSPLE----EDMSLFGDVFSEQDGVDFNTL    54

                 Strait jacket
    α1               Latency lasso           α2               β1      β1′
ProBMP9     LENVKVDFLRSLNLSGVPSQDKTRVEPPQYMIDLYNRYTSDKSTTPASNIVRSFSMEDAI   118
ProBMP10    LQSMKDEFLKTLNLSDIPTQDSAKVDPPEYMLELYNKFATDRTSMPSANIIRSFKNEDLF   114

 B1′        β2                  β3                  α3       β4
ProBMP9     SITATEDFPFQKHILLFNISIPRHEQITRAELRLYVSCQNHVDPSHDLKGSVVIYDVLDG   178
ProBMP10    SQPVS-FNGLRKYPLLFNVSIPHHEEVIMAELRLYTLVQRDRMIYDGVDRKITIFEVLES   173

                   β5            β6      α4                  β7
ProBMP9     TDAWDSATETKTFLVSQDI--QDEGWETLEVSSAVKRWVRSDSTKSKNKLEVTVESHR--   234
ProBMP10    KGDNEGERN-MLVLVSGEIYGTNSEWETFDVTDAIRRWQKSGSSTHQ--LEVHIESKHDE   230

                β9′           β10              α5
ProBMP9     ---KGCDTLDISVPPGSRNLPFFVVFSNDHSSGTKETRLELREMISHEQESVLKKLSKDG   291
ProBMP10    AEDASSGRLEIDTSAQNKHNPLLIVFSDDQSS-DKERKEELNEMISHEQLPELDNLGLDS   289

                                Furin cleavage site
ProBMP9     STEAGESSHEEDTDGHVAAGSTLARRKRSAGAGSHCQKTSLRVNFEDIGWDSWIIAPKEY   351
ProBMP10    FSSGPGEEALLQMRSNIIY--DSTARIRRNAKGNYCKRTPLYIDFKEIGWDSWIIAPPGY   347
                                 Furin cleavage site

ProBMP9     EAYECKGGCFFPLADDVTPTKHAIVQTLVHLKFPTKVGKACCVPTKLSPISVLYKDDMGV   411
ProBMP10    EAYECRGVCNYPLAEHLTPTKHAIIQALVHLKNSQKASKACCVPTKLEPISILYLD-KGV   406

ProBMP9     PTLKYHYEGMSVAECGCR         429
ProBMP10    VTYKFKYEGMAVSECGCR         424
```

**Supplementary Figure 5.** Sequence alignment of full-length human proBMP9 and proBMP10. The signal peptide is highlighted in cyan and GF-domain highlighted in yellow. Secondary structure elements, strait jacket and the latency lasso regions are marked over the sequence according to the sequence alignment in Mi *et al* [2], with grey lines showing those only observed in pro-TGFβ, not pro-BMP9. The red arrows indicate the furin-cleavage sites.

# Supplementary Figure 6

**(a)**

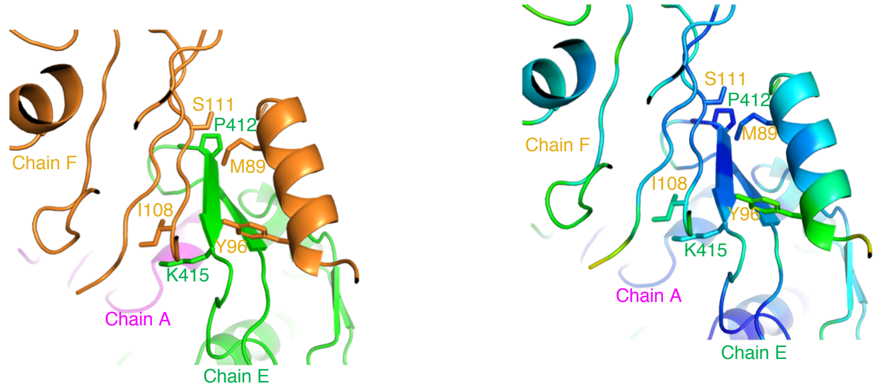


**(b)**

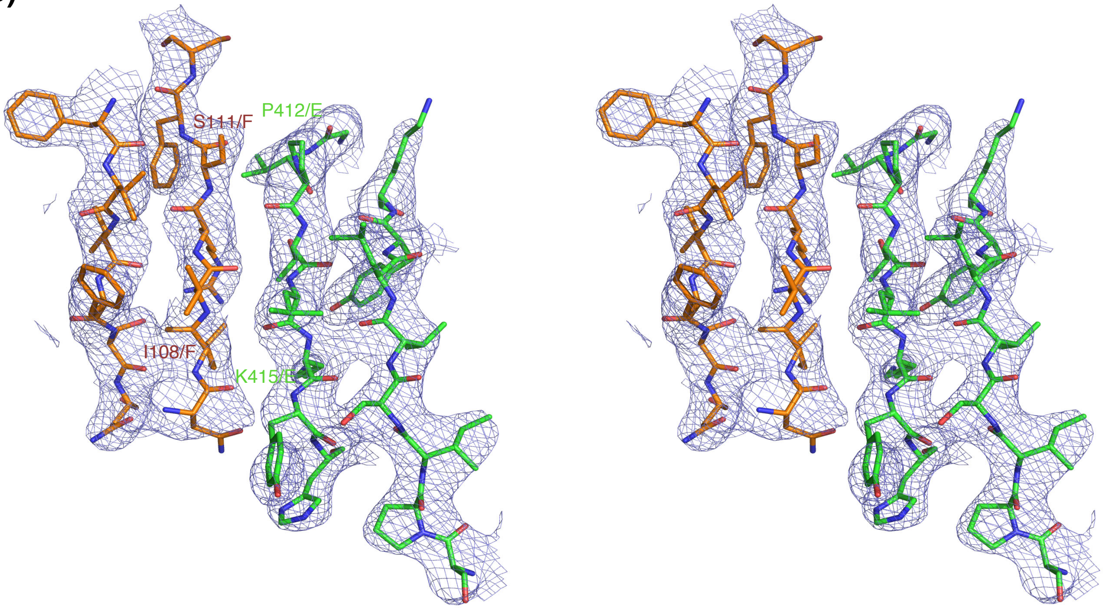


**Supplementary Figure 6.** Prodomain and GF-domain interface in the pro-BMP9:ALK1 structure. **(a)** Cartoon illustrations of the interface between prodomain and the GF-domain. Taken from identical view, left cartoon is coloured by different chains and right coloured by mainchain B factors. Chain A is ALK1, Chain E is BMP9 GF-domain and Chain F is the prodomain. Sidechains of prodomain M89, Y96, I108 and S111, and GF-domain P412 and K415 are shown to illustrate the boundaries of the secondary structures at the interface. **(b)** A stereo image of an electron density map from the same region of pro-BMP9:ALK1 structure. The final 2Fo-Fc electron density (grey, contoured at 1.5 σ) in the region of β-sheet interactions between BMP9 (green) and prodomain (orange), overlaid with the final refined model.

# Supplementary Figure 7

(a)

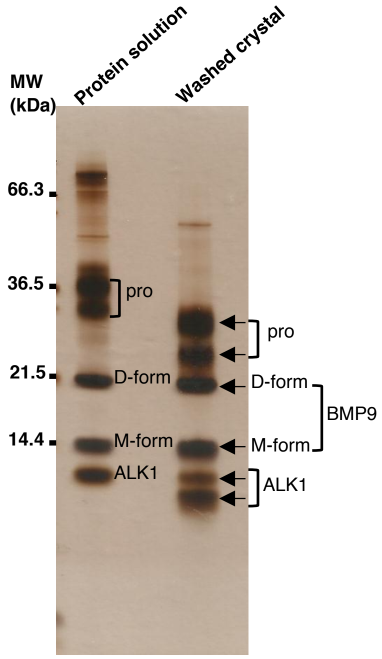


(b)

```
        10         20         30         40         50         60
MCPGALWVAL PLLSLLAGSL QGKPLQSWGR GSAGGNAHSP LGVPGGGLPE HTFNLKMFLE

        70         80         90        100        110        120
NVKVDFLRSL NLSGVPSQDK TRVEPPQYMI DLYNRYTSDK STTPASNIVR SFSMEDAISI

       130        140        150        160        170        180
TATEDFPFQK HILLFNISIP RHEQITRAEL RLYVSCQNHV DPSHDLKGSV VIYDVLDGTD

       190        200        210        220        230        240
AWDSATETKT FLVSQDIQDE GWETLEVSSA VKRWVRSDST KSKNKLEVTV ESHRKGCDTL

       250        260        270        280        290        300
DISVPPGSRN LPFFVVFSND HSSGTKETRL ELREMISHEQ ESVLKKLSKD GSTEAGESSH

       310        320        330        340        350        360
EEDTDGHVAA GSTLARRKRS AGAGSHCQKT SLRVNFEDIG WDSWIIAPKE YEAYECKGGC

       370        380        390        400        410        420
FFPLADDVTP TKHAIVQTLV HLKFPTKVGK ACCVPTKLSP ISVLYKDDMG VPTLKYHYEG

MSVAECGCR
```

**Supplementary Figure 7.** Sequence coverage in the pro-BMP9:ALK1 structure. ***(a)*** SDS-PAGE and silver staining of a washed pro-BMP9:ALK1 crystal. All bands in the washed crystal lane were cut out and subject to mass spectrometry peptide mapping. ***(b)*** proBMP9 sequence, with residues observed in mass spectrometry peptide mapping shown in red. Highlighted in cyan is the signal peptide and in yellow is the GF-domain.

# Supplementary Figure 8

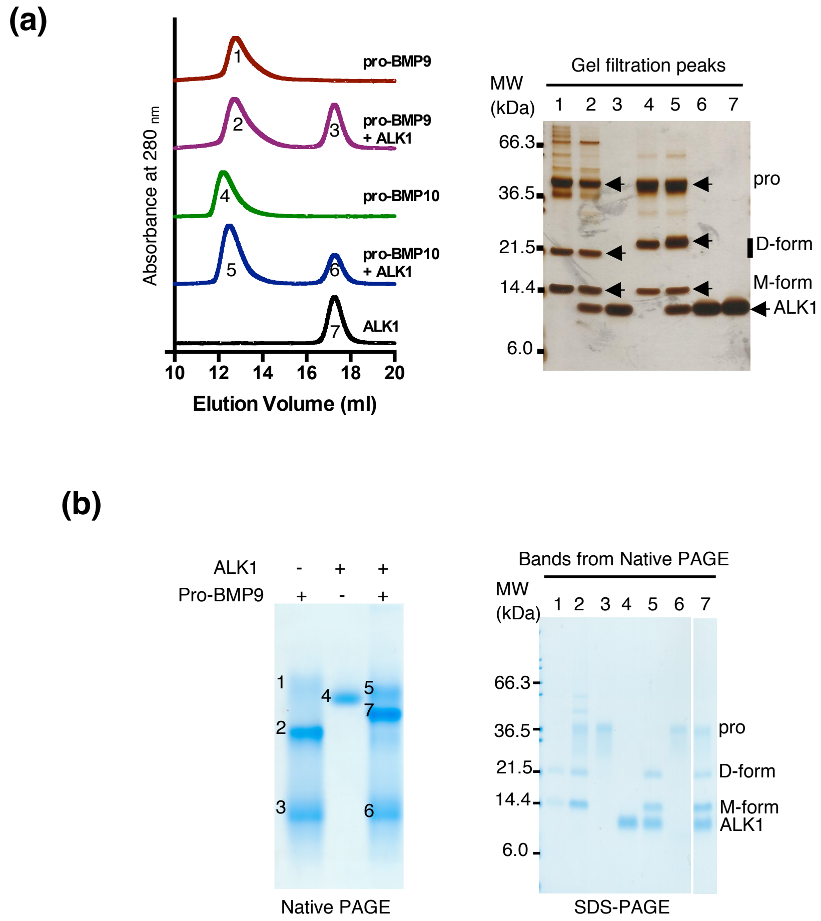


**Supplementary Figure 8.** ALK1 ECD can form complexes with pro-BMP9 and pro-BMP10 without displacing the prodomains. **(a)** Analysis of complex formation by analytical gel filtration. Purified pro-BMP9 or pro-BMP10 were run on an S200 10/300 gel filtration column, with or without being pre-mixed with a 2-fold molar excess of purified ALK1 ECD in TBS. The elution profiles were overlaid (left, only the region from 10 to 20 milliliters is shown). Fractions under peaks 1 to 7 were subject to a 12% non-reducing SDS-PAGE (right) and silver staining. The arrows highlight the prodomains (pro), GF-domain D-form and M-form [3], and ALK1 ECD. **(b)** Native PAGE analysis of complex formation between pro-BMP9 and ALK1. Bands on the native PAGE (left) were cut out and re-run on a non-reducing SDS-PAGE (right). Lanes 1 to 7 correspond to bands 1 to 7 on the native PAGE. Two parts of the same SDS-PAGE are shown, with the original uncropped gel provided in Supplementary Fig 11. Each sample has been subject to native PAGE experiment three times to ensure reproducibility.

# Supplementary Figure 9

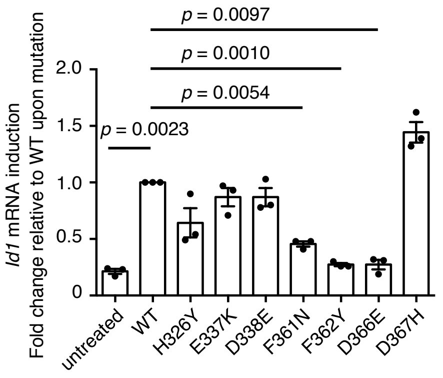


**Supplementary Figure 9.** *Id1* gene induction in C2C12 cells treated with pro-BMP9 or its mutants. C2C12 cells were seeded in 6-well plates overnight followed by serum starvation for 20 hours before the cells were treated with 4 ng/ml ligands (GF-domain concentration). Cells were harvested after 1-hour treatment followed by mRNA extraction and qPCR analysis. The qPCR primer sequences for the mouse genes are: *Id1* 5' ACGACATGAACGGCTGCTGCTACT and 5' GCTCACTTTGCGGTTCTGG; Beta-2-microglobulin 5' TGCTACTCGGCGCTTCAGTC and 5' AGGCGGGTGGAACTGTGTTAC. Data are presented as means ± SEM from three independent experiments. One-way ANOVA with Dunnett's post test against WT. Source data are provided as a Source Data file.

# Supplementary Figure 10

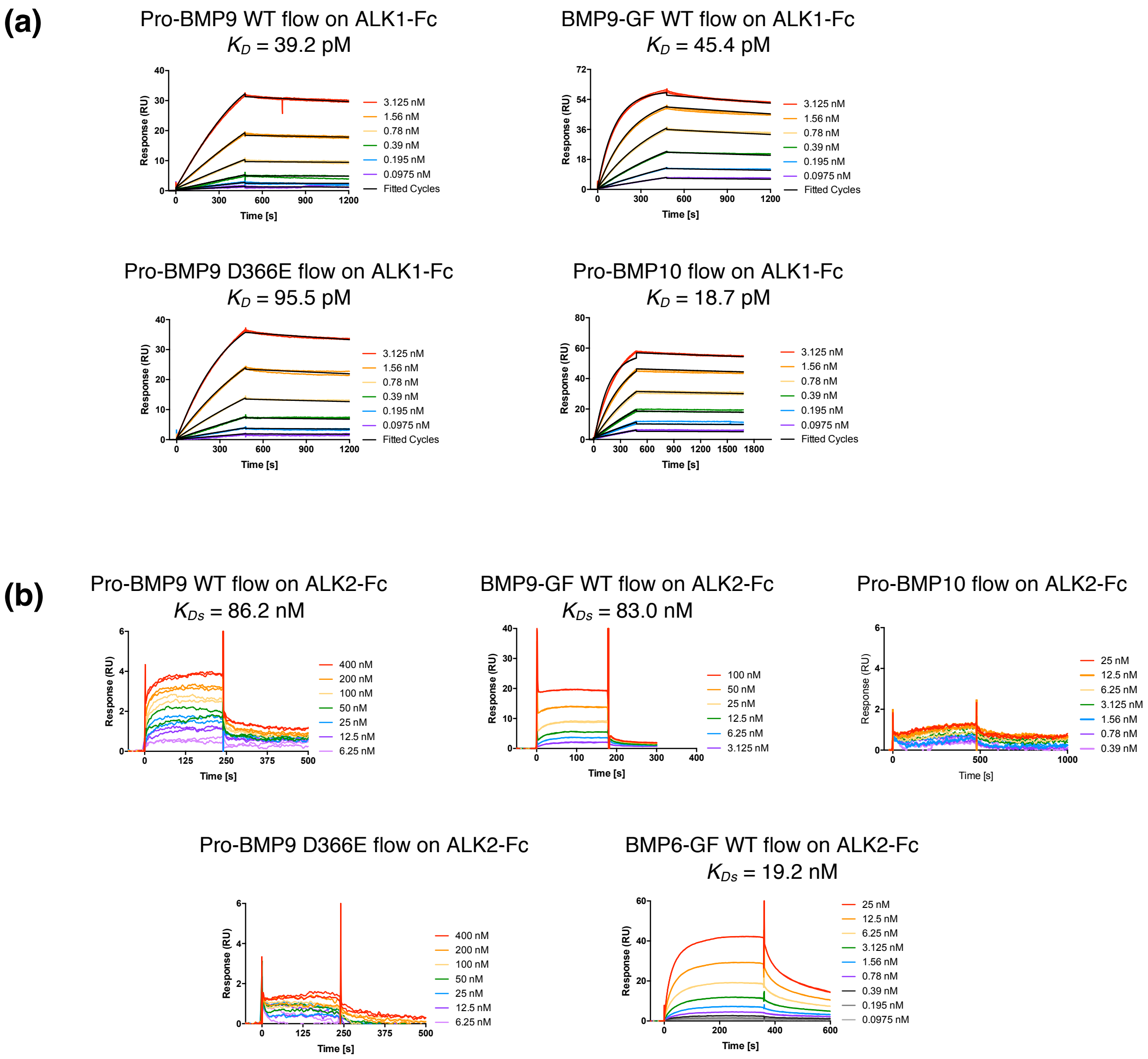


**Supplementary Figure 10.** Pro-BMP9 D366E mutation selectively lost binding to ALK2. **(a)** Pro-BMP9, BMP9-GF domain, pro-BMP9 D366E and pro-BMP10 binding on the ALK1-Fc surface. **(b)** Pro-BMP9, BMP9-GF domain, pro-BMP9 D366E and pro-BMP10 binding on the ALK2-Fc surface. A Biacore CM5 chip was coated with ALK1-Fc or ALK2-Fc (both from R&D Systems). Different BMP ligands were flowed over the chip at 40 µl/min at 25 ˚C on a Biacore T200 machine. The binding affinities were determined with either the build-in 1:1 binding model ($K_D$) or steady state fit ($K_{Ds}$) using the Biacore T200 Evaluation Software (Version 1.0). The exported curves were re-plotted in Prism. Control experiment using BMP6 GF-domain binding to ALK2-Fc demonstrates that the ALK2-Fc chip is fully functional.

# Supplementary Figure 11. Uncropped Original Gels and Blots

Fig 6f

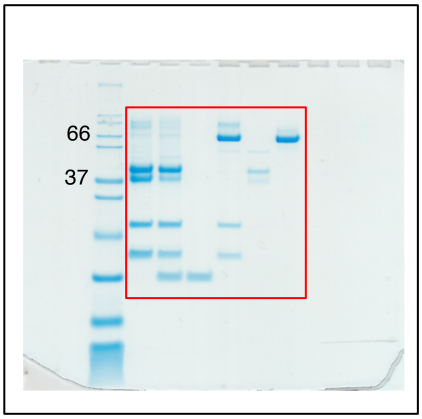


Supplementary Fig 8b

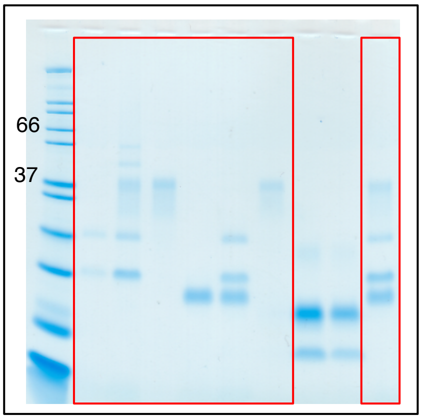


Fig 6g

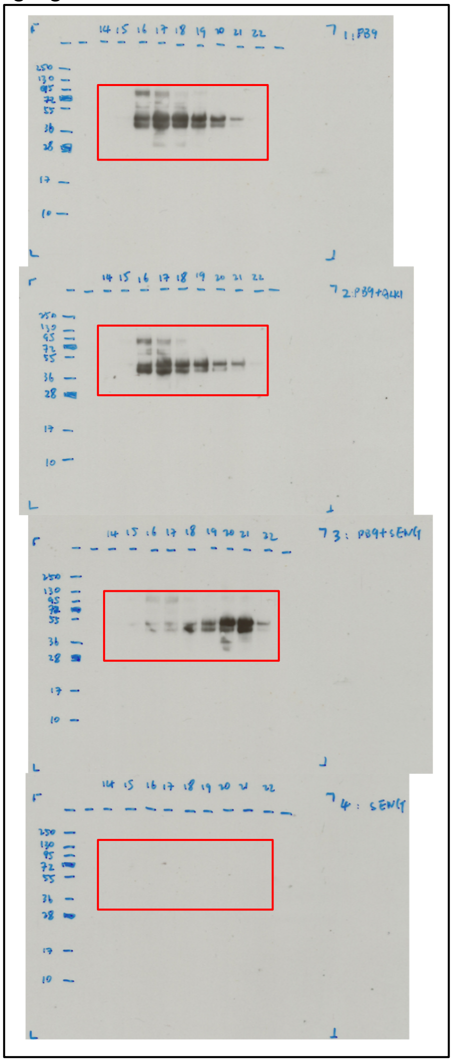

# Supplementary References

1. Townson, S. A. *et al.* Specificity and structure of a high affinity activin receptor-like kinase 1 (ALK1) signaling complex. *J. Biol. Chem.* **287**, 27313-27325 (2012).

2. Mi, L. Z. *et al.* Structure of bone morphogenetic protein 9 procomplex. *Proc. Natl. Acad. Sci. U. S. A.* **112**, 3710-3715 (2015).

3. Wei, Z., Salmon, R. M., Upton, P. D., Morrell, N. W. & Li, W. Regulation of Bone Morphogenetic Protein 9 (BMP9) by Redox-dependent Proteolysis. *J. Biol. Chem.* **289**, 31150-31159 (2014).
