# Supplementary material for: Molecular basis of ALK1-mediated signalling by BMP9/BMP10 and their prodomain-bound forms
Source: Nat Commun. 2020 Apr 1;11:1621. doi: 10.1038/s41467-020-15425-3 (PMC7113306; doi:10.1038/s41467-020-15425-3)
Supplement: Supplementary file 3 — Description of Additional Supplementary Files [file 41467_2020_15425_MOESM3_ESM.docx]

**Description of Additional Supplementary Files**

Supplementary Data 1.

Description: Microarray dataset 1. List of transcripts significantly up- and down-regulated by pro-BMP9 in human pulmonary artery endothelial cells after 1.5 hours treatment. Details of the experiments can be found in the Methods of the main article. Full dataset has been deposited to Gene Expression Omnibus, with the accession number of GSE134890.

Supplementary Data 2.

Description: Microarray dataset 2. List of transcripts significantly up- and down-regulated by pro-BMP10 in human pulmonary artery endothelial cells after 1.5 hours treatment. Details of the experiments can be found in the Methods of the main article. Full dataset has been deposited to Gene Expression Omnibus, with the accession number of GSE134890.
