# Supplementary material for: Molecular basis of ALK1-mediated signalling by BMP9/BMP10 and their prodomain-bound forms
Source: Nat Commun. 2020 Apr 1;11:1621. doi: 10.1038/s41467-020-15425-3 (PMC7113306; doi:10.1038/s41467-020-15425-3)
Supplement: Supplementary file 6 — Reporting Summary [file 41467_2020_15425_MOESM6_ESM.pdf]

nature research

Corresponding author(s): Wei Li
wl225@cam.ac.uk

Last updated by author(s): 28/02/2020

Reporting Summary

Nature Research wishes to improve the reproducibility of the work that we publish. This form provides structure for consistency and transparency in reporting. For further information on Nature Research policies, see Authors & Referees and the Editorial Policy Checklist .

Statistics

For all statistical analyses, confirm that the following items are present in the figure legend, table legend, main text, or Methods section.

n/a	Confirmed	
☐	☒	The exact sample size (*n*) for each experimental group/condition, given as a discrete number and unit of measurement
☐	☒	A statement on whether measurements were taken from distinct samples or whether the same sample was measured repeatedly
☐	☒	The statistical test(s) used AND whether they are one- or two-sided<br>*Only common tests should be described solely by name; describe more complex techniques in the Methods section.*
☒	☐	A description of all covariates tested
☒	☐	A description of any assumptions or corrections, such as tests of normality and adjustment for multiple comparisons
☒	☐	A full description of the statistical parameters including central tendency (e.g. means) or other basic estimates (e.g. regression coefficient) AND variation (e.g. standard deviation) or associated estimates of uncertainty (e.g. confidence intervals)
☒	☐	For null hypothesis testing, the test statistic (e.g. *F*, *t*, *r*) with confidence intervals, effect sizes, degrees of freedom and *P* value noted<br>*Give P values as exact values whenever suitable.*
☒	☐	For Bayesian analysis, information on the choice of priors and Markov chain Monte Carlo settings
☒	☐	For hierarchical and complex designs, identification of the appropriate level for tests and full reporting of outcomes
☒	☐	Estimates of effect sizes (e.g. Cohen's *d*, Pearson's *r*), indicating how they were calculated

Our web collection on statistics for biologists contains articles on many of the points above.

Software and code

Policy information about availability of computer code

Data collection	All X-ray diffraction data were collected at Diamond Light Source. Methods section of the manuscript contains detailed information of all the softwares used for data collection. All softwares are commercial or open source. The following versions of softwares were used for data collection: CCP4-7.0-macosx-x86_64, 7.0.044, phenix.refine (version 1.11.1 2575)
Data analysis	Methods section contains detailed information of all the softwares used for data analysis. All softwares are commercial or open source. The following versions of softwares were used: R (version 3.3), Oligo (version 1.40.2), limma package (version 3.32.1), Biacore T100 Evaluation Software (version 2.0.3) and Biacore T200 Evaluation Software (version 2.0).

For manuscripts utilizing custom algorithms or software that are central to the research but not yet described in published literature, software must be made available to editors/reviewers. We strongly encourage code deposition in a community repository (e.g. GitHub). See the Nature Research guidelines for submitting code & software for further information.

Data

Policy information about availability of data

All manuscripts must include a data availability statement. This statement should provide the following information, where applicable:

- Accession codes, unique identifiers, or web links for publicly available datasets
- A list of figures that have associated raw data
- A description of any restrictions on data availability

Coordinates and structure factors for all structures have been deposited to the Protein Data Bank, with the accession numbers of 6SF1, 6SF2 and 6SF3. Microarray data have been deposited to Gene Expression Omnibus, with the accession number of GSE134890. All additional experimental data are available from the corresponding author on request.

Field-specific reporting

Please select the one below that is the best fit for your research. If you are not sure, read the appropriate sections before making your selection.

☒ Life sciences ☐ Behavioural & social sciences ☐ Ecological, evolutionary & environmental sciences

For a reference copy of the document with all sections, see nature.com/documents/nr-reporting-summary-flat.pdf

Life sciences study design

All studies must disclose on these points even when the disclosure is negative.

Sample size	No sample-size calculations were performed. Sample sizes were chosen specifically for each experiments. For all signalling assays, we typically perform 3-5 independent experiment repeats to allow statistical analysis and robust conclusions to be drawn. For biochemical gel analysis which are not quantitative, at least two repeats were performed to confirm the observation. For Microarray analysis, the sample size was determined by minimum requirement for statistical analysis (3 samples), one backup sample and the affordability of the assays, hence N=4 was chosen. For in vivo heterotopic (HO) bone formation assay, because the goal is not to obtain quantitative comparison, but to provide a solid evidence whether yes or no for HO bone formation, we therefore performed minimum N=4 injections for each condition. Control samples, such as negative treatment, WT proteins, were included in each experiment with additional N numbers to allow robust controls.
Data exclusions	No data exclusions in this manuscript.
Replication	Replicate experiments were successful. For quantitative measurements, three or more independent experiments were carried out and statistical analysis performed. For non-quantitative biochemical assays, each experimental condition was repeated at least one more time to confirm the observation. X-ray structural coordinates were checked by MolProbity server before deposition to Protein Data Bank and independent validation reports obtained from Protein Data Bank.
Randomization	Mice were randomized to treatment group from aged matched littermates. For all the other quantitative studies, randomization is not relevant because all the cells were under identical conditions before different treatment samples applied.
Blinding	The investigator was blinded in the in vivo heterotopic bone-forming experiment in mice. Microarray data collection were blinded until bioinformatic analysis. For other signalling experiments, blinding is not relevant because the operators cannot predict the results from raw data without several steps of data processing which take at least two days.

Reporting for specific materials, systems and methods

We require information from authors about some types of materials, experimental systems and methods used in many studies. Here, indicate whether each material, system or method listed is relevant to your study. If you are not sure if a list item applies to your research, read the appropriate section before selecting a response.

Materials & experimental systems

n/a	Involved in the study	
☐	☒	Antibodies
☐	☒	Eukaryotic cell lines
☒	☐	Palaeontology
☐	☒	Animals and other organisms
☐	☒	Human research participants
☒	☐	Clinical data

Methods

n/a	Involved in the study	
☒	☐	ChIP-seq
☒	☐	Flow cytometry
☒	☐	MRI-based neuroimaging

Antibodies

Antibodies used	phospho-Smad1/5 (Ser463/465)(41D10) Rabbit mAb, #9516, Cell Signaling Technology; Anti-Alpha-tubulin mouse monoclonal, clone DM1A, #T6199, Sigma Aldrich.
Validation	phospho-Smad1/5: species cross-reactivity is determined by western blot, specificity/sensitivity is determined by flow cytometric analysis. Details can be found at company website.<br>Anti-Alpha-tubulin information can be found at company website: https://www.sigmaaldrich.com/catalog/product/sigma/t6199?lang=en&region=GB&cm_sp=Insite-_-prodRecCold_xviews-_-prodRecCold3-1

Eukaryotic cell lines

Policy information about cell lines

Cell line source(s)	Human pulmonary artery endothelial cells (hPAECs) were purchased from Lonza (Cat. No. CC-2530), C2C12 cells (cat. No. CRL-1772) and HEK EBNA cells (cat. No. CRL-10852) were from ATCC, human pulmonary artery smooth muscle cells (hPASMCs) were isolated in house and have been characterized and published previously.
Authentication	hPAECs, C2C12 and HEK EBNA cells were authenticated by Lonza and ATCC, respectively. The isolation and authentication of hPASMCs have been reported in ref 59 of the manuscript, also described in the Methods section. The authentification of PASMCs including the source of vessels for isolating the cells, morphology of the cells and positive calponin staining.
Mycoplasma contamination	All cells tested negative for mycoplasma
Commonly misidentified lines (See ICLAC register)	No cell lines used are listed in the database of commonly misidentified cell lines.

Animals and other organisms

Policy information about studies involving animals; ARRIVE guidelines recommended for reporting animal research

Laboratory animals	Male C57/BL6 mice of 7-8 weeks' old
Wild animals	No wild animals used in this study
Field-collected samples	No field-collected samples in this study
Ethics oversight	Ethical approval was obtained from the University of Cambridge's Animal Welfare and Ethical Review Board

Note that full information on the approval of the study protocol must also be provided in the manuscript.

Human research participants

Policy information about studies involving human research participants

Population characteristics	The clinical information on the three subjects are: 68-year old male with carcinoid tumour, 57-year old female with emphysema and 64-year old female with squamous mataplasia. Such information was not relevant to the conclusion of the paper hence was not used in the manuscript.
Recruitment	PASMCs were isolated from the lung resection specimens from the Papworth Hospital Research Tissue Bank. Informed consents were obtained from all donors.
Ethics oversight	Ethical approval was obtained from the NRES Committee East of England -Cambridge East (reference number: 18/EE/0269).

Note that full information on the approval of the study protocol must also be provided in the manuscript.
